# Alternative Vaccination Routes against Paratuberculosis Modulate Local Immune Response and Interference with Tuberculosis Diagnosis in Laboratory Animal Models

**DOI:** 10.3390/vetsci7010007

**Published:** 2020-01-10

**Authors:** Rakel Arrazuria, Iraia Ladero, Elena Molina, Miguel Fuertes, Ramón Juste, Miguel Fernández, Valentín Pérez, Joseba Garrido, Natalia Elguezabal

**Affiliations:** 1 Animal Health Department, NEIKER-Instituto Vasco de Investigación y Desarrollo Agrario. Derio, E-48160 Bizkaia, Spain; raarrazu@utmb.edu (R.A.); iladero@neiker.eus (I.L.); emolina@neiker.eus (E.M.); mfuertes@neiker.eus (M.F.); rajuste@serida.org (R.J.); jgarrido@neiker.eus (J.G.); 2 Department of Animal Health, Instituto de Ganadería de Montaña (CSIC-ULE), Facultad de Veterinaria, Universidad de León, E-24071 Leon, Spain; m.fernandez@unileon.es (M.F.); valentin.perez@unileon.es (V.P.)

**Keywords:** *Mycobacterium avium* subsp. *paratuberculosis*, vaccination routes, rabbit model, macrophage polarization, skin test

## Abstract

Paratuberculosis (PTB) is an enteric granulomatous disease caused by *Mycobacterium avium* subsp. *paratuberculosis* (MAP) that mainly affects ruminants. Current vaccines have shown to be cost–effective control reagents, although they are restricted due to cross-interference with bovine tuberculosis (bTB). Therefore, novel vaccination strategies are needed and this study is focused on evaluating alternative vaccination routes and their effect on the local immune response. The MAP oral challenge rabbit model was used to evaluate and compare an experimental inactivated MAP vaccine through oral (VOR) and intradermal (VID) routes. The VID group presented the highest proportion of animals with no visible lesions and the lowest proportion of animals with MAP positive tissues. Immunohistochemistry analysis revealed that the VID group presented a dominantly M1 polarized response indicating an ability to control MAP infection. In general, all vaccinated groups showed lower calprotectin levels compared to the non-vaccinated challenged group suggesting less active granulomatous lesions. The VID group showed some degree of skin test reactivity, whereas the same vaccine through oral administration was completely negative. These data show that PTB vaccination has an effect on macrophage polarization and that the route influences infection outcome and can also have an impact on bTB diagnosis. Future evaluation of new immunological products against mycobacterial diseases should consider assaying different vaccination routes.

## 1. Introduction

Paratuberculosis (PTB), also named Johne’s disease, is a chronic granulomatous enteritis produced by *Mycobacterium avium* subsp. *paratuberculosis* (MAP). Although ruminants are the most affected species, MAP has been detected in a wide range of domestic and wildlife animals [1,2,3]. In addition, a huge body of literature links MAP to human’s Crohn’s disease [4,5,6] and efforts are being made to definitely clarify this controversial issue [7]. 

PTB causes economic losses to the livestock industry worldwide [8]. Current control programs favor test and cull strategies that are expensive and inefficient due to the lack of sensitivity of current diagnostic techniques. To date, vaccination with whole cell inactivated vaccines has shown to be the best cost-effective method for PTB infection control [9] when combined with management interventions since it can reduce the incidence of MAP shedding and manifestation of clinical signs. Although MAP vaccination does not completely prevent the infection, it is recommended as a PTB control method in small ruminants [10]. In cattle, PTB vaccination has been shown to reduce MAP shedding [11,12,13], improve milk production [13] and it has also been associated with a longer productive life [11]. Despite these beneficial traits, the use of available commercial vaccines in cattle is limited or not allowed in many countries due to the interference with bovine tuberculosis (bTB) official diagnostic tests. Recent studies have suggested that this drawback could be overcome by introducing variations in the official tests [14], but this depends on legislative modifications that do not seem implementable in the near future.

In consequence, effort is focused on developing new vaccines that should be more effective at inducing protection than current commercial vaccines and that should not cross-react or interfere with bTB diagnosis. Several immunogenic products under study include live attenuated vaccines [15], subunit vaccines [16], probiotics like Dietzia [17], DNA vaccines [18], and recombinant proteins [19]. Alternative to oil-in-water adjuvants are also being explored [20], and recent studies have revealed that not only the antigen is important for effective immunization or avoiding cross-reactivity but also the vaccination route plays an important role on the outcome. The *Mycobacterium bovis* (Mbv) vaccination route has shown an effect on interference in bTB diagnosis in wild boar [21] and cattle [22], where the intramuscular route showed a clear and consistent bovine purified protein derivative (PPD) IFN-γ dose dependent response, but no positive reactors were detected in the animals vaccinated by the oral route. Recently, it has been reported that oral vaccination with whole cell inactivated Mbv did not interfere with the ante-mortem diagnosis for tuberculosis detection in goats [23], suggesting that the vaccination route could also have an impact on bTB official diagnostic testing. 

Not many studies have assessed the oral route for vaccination against PTB although it would more closely mimic natural exposure to MAP and probably activate mucosal immunity. The few reported studies include oral subunit vaccination in a goat model [24] and whole cell inactivated vaccination in sheep [25,26]. Unsuccessful results could be due to inadequate dosing, absence of adjuvant, or the fact that subunit vaccines are directed against a restricted number of antigens limiting a protective immune response.

In recent years, there has been a revived interest in the intradermal vaccination route, as a result of the development of innovative technologies in micro needle design or needle free injection devices, aiding for a more precise and easier inoculation. Intradermal vaccination has been shown to be more effective than intramuscular and subcutaneous vaccination against many pathogens [27,28]. Surprisingly, reports on the effect of intradermal vaccination route against MAP infection are inexistent in the literature. 

Antigen presenting cells present in different locations can generate different kinds of T cell responses [29] indicating that different vaccination routes can lead to diverse outcomes or in other words that the route can modulate the immune response. Macrophages play a critical role in the host-pathogen interaction in PTB [30] and these immune cells are characterized by a remarkable diversity and plasticity and can acquire distinct functional phenotypes depending on polarization status leading to different types of immune responses [31]. 

In this context, the main objective of the present study was to evaluate a whole cell inactivated vaccine administered through the intradermal and oral routes for MAP protection and effect on macrophage polarization in lesions in the rabbit MAP infection model, and for interference with bTB diagnosis in the guinea pig model. 

## 2. Materials and Methods 

### 2.1. Vaccination and Challenge Experiment

#### 2.1.1. Experimental Design

New Zealand white female rabbits of 7 weeks of age (n = 25) were purchased from authorized experimental animal dealers (Granja San Bernardo, Tulebras, Spain). Upon arrival at NEIKER facilities (Derio, Spain), animals were randomly allocated into five experimental groups with 5 animals each: non-infected control group (NIC); infected control group (IC); experimental intradermal vaccine group (VID); experimental oral vaccine group (VOR); and control vaccine group (VC) and left on a 15 days adaptation period being fed with weaning growth pellets *ad-libitum* until the age of 12 weeks. Afterwards, animal feed was limited to 30–35 g of dry matter/kg bodyweight per day whereas water was available *ad-libitum*.

Intradermal and oral vaccinations were carried out with an experimental whole-cell MAP strain chemically inactivated vaccine that does not include any adjuvant in its formulation. As a control, subcutaneous vaccination was performed with Silirum^®^ (CZV, Porriño, Pontevedra, Spain), a whole-cell inactivated commercial vaccine that carries a highly refined mineral oil adjuvant, that is approved for its use in cattle in some countries and that is the current standard for vaccination, being an appropriate control. 

On day 1, vaccination was performed in the corresponding groups (VID, VOR, VC) with 1 mL of 12.5 mg antigen (calculated by the wet weight method to be 2.3 × 10^7^ CFU). Fifteen days after, a second dose was administered to the VID and VOR groups. VID and VC vaccination were performed distributing the dose in two shots performed on the back. VID was administered using a 29G needle on two previously shaved sections and VC using a 25G needle. VOR vaccine was administered via needle-less syringe. Afterwards, on three consecutive days (day 26, 27, and 28) all animals except the ones belonging to the NIC group were orally challenged via needle-less syringe with a dose of 10^9^ CFU per day of MAP strain K10 obtained as described previously [32]. 

Weight recording and blood sampling was done at different time points (S0–S6). Blood was centrifuged at 1500× *g* for 10 min RT to obtain plasma which was stored at −20 °C for further analysis. All animals were euthanized at the end point (195–200 days) with an intracardiac pentobarbital dose (200 mg/kg) after deep sedation with xylazine (5 mg/kg) and ketamine (35 mg/kg). Immediately afterwards, a complete necropsy was performed focusing on the digestive system. The observed gross pathology was scored as follows: Normal appearance (0), mild thickening of ileum and/or distal jejunum (1), moderate ileum and/or jejunum thickening plus lymphangiectasia (2). Presence of white spots corresponding to caseous necrosis foci in sacculus rotundus (SR) and/or vermiform appendix (VA) (3). Presence of white spots in SR and/or VA plus ileum and/or jejunum thickening or lymphangiectasia (4). Thereafter, tissue samples of SR, VA, mesenteric lymph node (MLN), liver and tonsil were collected and the size of the SR, VA, and MLN was noted.

#### 2.1.2. Tissue Gene Extraction and MAP PCR

Collected tissues (SR and VA) stored at −20 °C were thawed overnight at 4 °C and tissue DNA was extracted with DNA Extract-VK (Vacunek S.L, Derio, Spain) according to the manufacturer’s instructions and as described previously [32]. Extracted DNA was stored at −20 °C until assayed. A real time multiplex PCR detecting IS900 and ISMAP02 DNA sequences of MAP was performed with 350 ng/μL as described by Sevilla et al. [33]. PCR results were analyzed using the 7500 System SDS software v. 1.4 (Applied Biosystems, Alcobendas, Spain). Threshold cycle (C*_T_*) and baseline were automatically determined by the software and verified by visual examination of the threshold line in amplification plots. C*_T_* values equal or below 38 for both *IS900* and *ISMAP02* probes were considered positive, C*_T_* values over 38 for both targets probe and under 38 for IAC probe were considered negative. 

#### 2.1.3. PPA-3 Enzyme Linked Immunosorbent Assay (ELISA)

To assess humoral immunity, an in-house indirect ELISA was performed using paratuberculosis protoplasmic antigen 3 (PPA-3) (Allied Lab, Fayette, MO, USA) as described previously [34]. Briefly, microtiter plates were coated with 100 μL of PPA-3 (0.04 mg/mL) diluted in 0.5% sodium carbonate buffer (pH 9.6) and incubated at 4 °C overnight. After coating, plates were washed once with wash solution (0.05% Tween 80/0.85% sodium chloride). Plasma samples were thawed, adsorbed with *Mycobacterium phlei* in saline solution (5 g/L) diluted 1:100 in 0.05% Tween 80 in PBS (PBS-T), added to coated plates (100 μL/well) and incubated 2 h at RT in a humid chamber. Following incubation plates were washed three times with wash solution (300 μL/well). Recombinant protein G peroxidase (Sigma-Aldrich, St. Louis, MO, USA) 0.025 μg/mL in PBS-T was added (100 μL/well) and plates were incubated at RT for 2 h. The plates were washed again three times with wash solution (300 μL/well) and then peroxidase substrate (0.01% ABTS, Sigma Aldrich, St. Louis, MO, USA) was added and plates were further incubated at RT for 30 min in darkness. The reaction was stopped by the addition of 2% hydrofluoric acid (100 μL/well). The absorbance was measured at 405 and 450 nm using an automated ELISA plate reader (Multiskan EX^®^, Thermo Lab Systems, Helsinki, Finland). The reading obtained at 450 nm was subtracted from the reading of 405 nm and results were expressed as an ELISA index calculated by dividing the sample mean absorbance by the negative control mean absorbance. Optical density (OD) values were normalized across plates using the following calculation: ELISA index = (Mean sample OD) × (Mean OD of all positive control plates/Mean OD of the positive control plate).

#### 2.1.4. Histopathology

Samples from SR and VA, mesenteric lymph node, liver and tonsil were collected and fixed in 10% neutral buffered formalin solution for a minimum of 24 h, trimmed and dehydrated through graded alcohols. Afterwards, samples were embedded in paraffin wax and sectioned at 3–5 μm. These sections were mounted on glass slides and stained with haematoxylin and eosin (HE) according to standard procedures. Slides were examined blindly by an experienced pathologist giving an histopathology score. Severity of the lesions were graded as 0 if absent, and from 1 to 3 if present based on the number and extent of granulomatous lesions. Those graded as 1 were characterized by the presence of few granulomas, composed of about 50–60 macrophages, only in the interfollicular areas of the intestinal lymphoid tissue. In the other 2 categories, granulomatous lesions were seen affecting the lymphoid tissue and intestinal lamina propria with different intensity. 

#### 2.1.5. Immunohistochemistry

For immunohistochemistry analysis sections of 3–5 μm were placed on poly-l-lysine coated slides and immunohistochemically stained using the Envision + System (Dako, Agilent Technologies, Glostrup, Denmark). Different monoclonal antibodies raised against antigens expressed by macrophages, including M1 and M2 subpopulations, and proliferation markers were used (Table 1).

Briefly, sections were deparaffinized and antigen retrieval was performed in the PT Link system (Dako, Agilent Technologies, Glostrup, Denmark) at 96 °C using a pH 6 or pH 9 retrieval solution (Table 1). After hydration, the sections were incubated in 3% hydrogen peroxide in methanol for 30 min to eliminate endogenous peroxidase. Rehydrated slides were rinsed in PBS of pH 7.4, and sections were incubated with the primary antibodies diluted in PBS (Table 1) overnight at 4 °C in a humidified chamber. After washing in PBS, sections were incubated for 40 min at room temperature with EnVision + horseradish peroxidase solution (Dako, Agilent Technologies, Santa Clara, CA, USA) for the monoclonal antibody. After washing in PBS, antibody localization was determined using 3,3-diaminobenzidine (Sigma-Aldrich Corp., Madrid, Spain). Sections were counterstained with Mayer’s hematoxylin for 10 s. Slides were mounted with DPX (dibutyl phthalate xylene) and observed under a light microscope. Appropriate species and isotype-matched immunoglobulins were used as negative controls. 

For this study, only those immunolabeled cells with a clear macrophage morphology (abundant cytoplasm and ovoid nucleus) or lymphocyte morphology in the case of IFN-γ analysis that were forming part of the granulomas present in the different lesion types were considered and evaluated. In each slide, 20 representative fields containing granulomatous lesions were selected, photographed and analyzed. Cell counting and image analysis were performed using the Image J processing and analysis software (US National Institutes of Health, Bethesda, MD, USA).

For staining comparison among animals, samples were scored semi-quantitatively using a complete immunohistochemistry-score (IHC-score) that considers both the staining intensity and the percentage of positively immunolabeled cells as described previously [37]. Briefly, for each type of lesion, the IHC-score was calculated by adding the products of the percentage of cells (0–100) labeled at a given staining intensity present in each selected field and the staining intensity score (0, none; 1, weak; 2, moderate; and 3, intense). Thus, the IHC-score obtained in each random field could range between 0 and 300.

### 2.2. Interference with bTB Diagnosis

Dunkin Hartley female guinea pigs of 4 weeks of age (n = 9) were purchased from authorized experimental animal dealers (Envigo, Barcelona, Spain). Upon arrival at NEIKER facilities (Derio, Spain), animals were randomly allocated into three experimental groups with 3 animals each: experimental intradermal vaccine group (VID), experimental oral vaccine group (VOR); and controlvaccine group (VC). Animals were fed guinea pig growing pellets and water ad-libitum throughout the experiment. After a 15 day adaptation period, groups VID and VOR were vaccinated. VID animals received intradermal injections of the experimental whole-cell MAP inactivated vaccine in two sites of their right flank, whereas VOR animals received the experimental whole-cell MAP inactivated vaccine orally. 15 days after, both groups received a second dose in identical conditions and the VC animals were vaccinated subcutaneously with the commercial vaccine (Silirum^®^). All vaccine doses were composed of 12.5 mg of antigen measured by wet weight [38]. After 5 weeks the left flank of all animals was shaved and treated with depilatory crème and three intradermal injections of 100 μL of saline, 1:100 dilution of avian PPD (A PPD) (CZ Veterinaria, Porriño, Spain) and 1:100 bovine PPD (B PPD) (CZ Veterinaria, Porriño, Spain) following a Latin square pattern were applied. The diameters of the reddish circles that form around the injection sites (delayed type hypersensitivity reaction) were measured after 48 h. Animals were euthanized on day 53 by intracardiac pentobarbital (200 mg/kg) injection after deep sedation with ketamine (50 mg/kg) and xylazine (5 mg/kg). 

### 2.3. Statistical Analysis

To test data normal distribution Shapiro–Wilk test was applied. Data normally distributed (weight gain percentage: calculated as the percentage of the initial weight, size of SR, VA and MLN) were analyzed by one-way analysis of variance (ANOVA) test for route as the independent variable, followed by a Dunnett’s multiple comparison test to compare all groups against the control group and Tukey post-hoc test to compare among the different groups. A two-way repeated measures ANOVA was applied to test differences in ELISA index values among groups at different sampling points.

The histopathology index was calculated as the sum of the scores per tissues divided by the number of examined tissues. Non-normally distributed data (gross pathology score, sum of all tested tissues, histopathology score, IHC-score) were tested using the non-parametric Kruskal-Wallis test, followed by Dunn’s post-hoc test for multiple comparisons among groups.

Figures were generated using GraphPad Prism 7 (GraphPad Software Inc., La Jolla, San Diego, CA, USA), Tableau software and Microsoft Excel 2010 while R statistical software was used for data analysis. For all tests a probability value of <0.05 was considered significant.

## 3. Results

### 3.1. Vaccination and Challenge Experiment

#### 3.1.1. Weight Gain, Gross Pathology and MAP Detection

**Figure 1:**
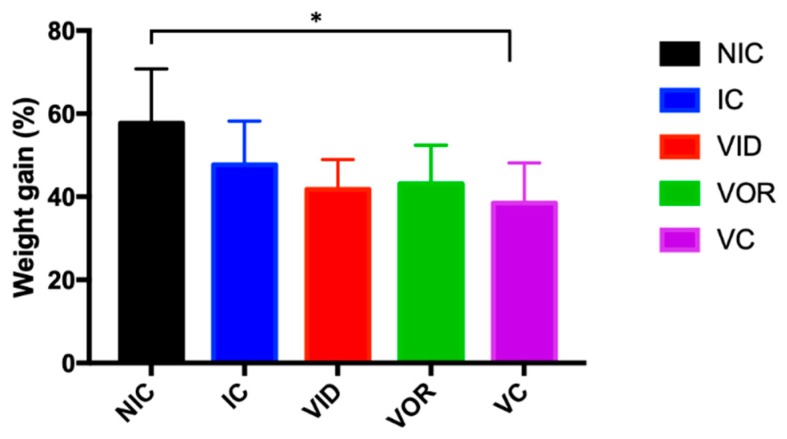
Weight gain during experimental period. Bars represent the mean and the error bars the standard error of the mean (SEM). * Significant differences were detected by Dunnet’s post hoc between the non-infected control group (NIC) and the control vaccine (VC) group (*p* = 0.024).

Weight gain during experimental period calculated as the percentage of weight gain in relation to the initial weight, was significantly higher (*p* = 0.024) in animals from the NIC group compared to the VC group (Figure 1).

At necropsy, lesions compatible with PTB infection consisting of mild and moderate ileum and distal jejunum thickness, intestinal and mesentery lymphangiectasia and presence of white spots corresponding to caseous necrosis foci in SR and VA were observed (Figure S1). These lesions were present in 60% of animals belonging to the IC group, 40% of the VID group and 80% of VOR and VC groups (Table 2). MAP was detected by PCR in 80% of animals of the IC, VOR, and VC groups and in 40% of animals belonging to the VID group (Table 2). 

#### 3.1.2. Humoral Response

**Figure 2:**
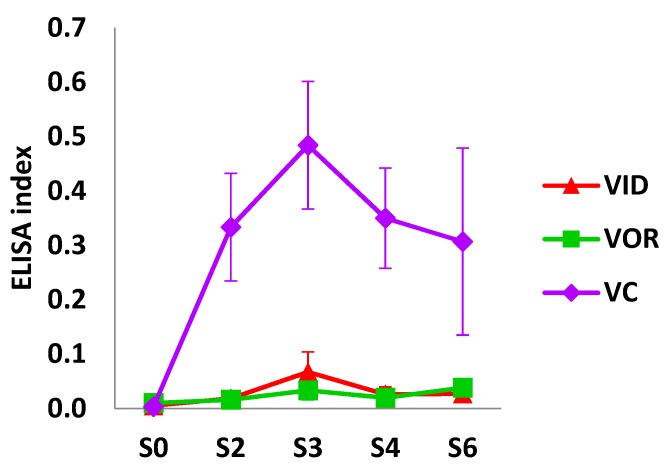
ELISA index time course of the vaccinated animal groups.

Humoral immune response measured by ELISA and an index was calculated during the experiment is represented on Figure 2. As expected, the humoral immune response did not show any difference between groups at the baseline sampling (S0). NIC and IC animals did not show an antibody level increase throughout experiment samplings (data not shown). At day 55 (S2), animals of the VC group showed an increase in anti-PPA-3 antibody levels. At this point, the ELISA index was significantly higher in VC group compared to the experimental vaccine groups (VID, VOR) (*p* < 0.0001). VC animals continued with significantly higher antibody levels after 90 days (S3) and 115 days (S4) of the beginning of the experiment. In these two samplings points, all pairwise comparisons involving the VC group yielded a *p*-value < 0.0001. When sampling period increments representing crucial experimental interventions such as vaccination (S2 − S0), vaccination plus challenge (S3 − S0) and the end of the experiment compared to the beginning (S6 − S0) were analyzed, the VC group showed highest antibody values and significant differences compared to the other vaccinated groups in periods (S2 − S0; vaccination) and (S3 − S0; vaccination + challenge). In the last sampling point (S6), after 160 days of the beginning the experiment, the VC group showed a decrease in PPA-3 antibody levels, although these were still higher than in the rest of the experimental groups (*p* < 0.001). 

Infected controls (IC), experimental intradermal vaccine animals (VID), experimental oral vaccine animals (VOR), control vaccine (VC) animals. Sacculus rotundus (SR) and vermiform appendix (VA). Histopathology score: 0 if no lesion, and from 1 to 3 if present based on the number and extent of granulomatous lesions. Gross pathology score: normal appearance (0), mild thickness of ileum and/or distal jejunum (1), moderate ileum and/or jejunum thickness plus lymphangiectasia (2), presence of white reactive spots in SR and/or VA (3), presence of white reactive spots in SR and/or VA plus ileum and/or jejunum thickness or lymphangiectasia (4).

In the VC group vaccination site nodules were measured showing variability in size which was expressed as cm^2^. Vaccination site nodule size was positively correlated to anti-PPA-3 antibody levels in the VC group (Pearson: 0.935, *p*: 0.02) (Figure S2).

#### 3.1.3. Histology

Granulomatous lesions were detected in SR and VA in all infected groups. The histology score of these two lymphoid tissues is detailed in Table 2. 

**Figure 3:**
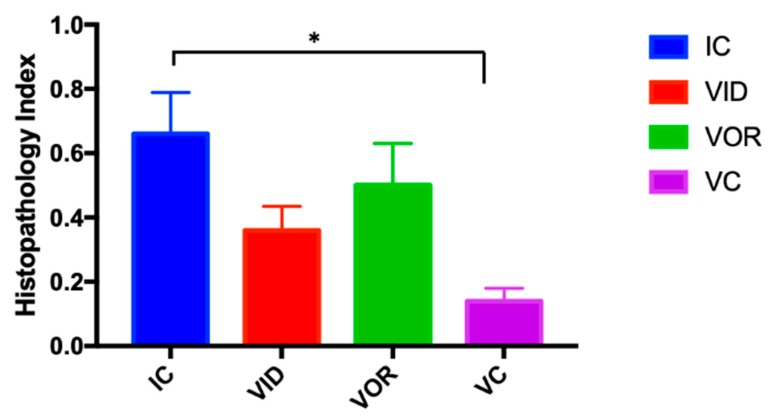
Histopathology index calculated as the sum of the scores per tissues divided by the number of examined tissues of the experimental groups. Error bars represent the standard error of the mean. * *p* < 0.05.

The IC group presented the highest number of microscopically affected tissues (SR, VA, tonsils, liver and MLN) followed by the VOR group, that did not show lesions in liver. Interestingly, the VID group showed lesions in liver but not in mesenteric lymph nodes in contrast to the VOR group. Finally, the VC group was the one with the lowest number of affected tissues. The histopathology index was significantly lower in the VC group compared to the IC group (*p* = 0.011) suggesting a healing or protective capacity of VC (Figure 3).

Vaccination site skin histology of VID animals showed a small necrotic area with dispersed acid-fast bacilli (Figure S2).

#### 3.1.4. Cell Subsets on Granulomatous Lesions

**Figure 4:**
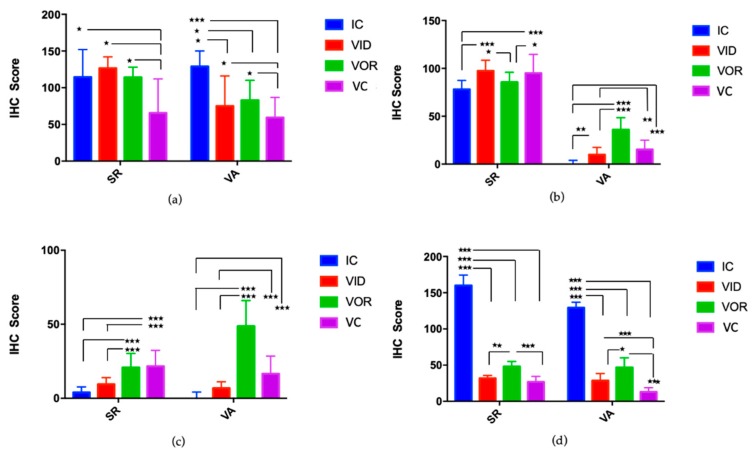
Macrophage polarization status. Mean immunohistochemistry score (IHC-score) in sacculus rotundus (SR) and vermiform appendix (VA) in the infected controls (IC), experimental intradermal vaccine (VID), experimental oral vaccine (VOR), and control vaccine (VC) animals for (**a**) IFNγ; (**b**) TNFα; (**c**) CD163; (**d**) calprotectin. Error bars represent the Confidence Interval of 95%. * *p* < 0.05; ** *p* < 0.01; *** *p* < 0.001.

Representative micrographs of the immunohistochemical characterization of SR granulomatous lesions are shown on Figure S3. The calculated IHC score for the selected markers on the granulomatous lesions of SR and VA are shown on Figure 4. There were less IFN-γ positive cells in the granulomatous lesions of SR in the VC group compared to the VID, VOR and IC groups (*p* = 0.013, *p* = 0.046, *p* = 0.023, respectively) (Figure 4a). The same pattern of IFN-γ detection among study groups was observed in VA, where VC presented less cells immunolabelled for IFN-γ compared to VID, VOR and IC (*p* = 0.016, *p* = 0.012 and *p* < 0.001, respectively). In addition, in the VA the IC group presented significantly higher IFN-γ IHC scores compared to VID and VOR (*p* = 0.010 and *p* = 0.013).

The IHC score for TNF-α (Figure 4b) in the granulomatous lesions of the SR showed lower values in the IC group compared to VID and VC (*p* < 0.001 and *p* < 0.001), and in VOR comparing with VID and VC (*p* = 0.013 and *p* = 0.021). In the VA, the levels of this marker were much lower than in the SR and again the IC group presented lower values than VID and VC (*p* = 0.002 and *p* < 0.001) and the VOR group was the one that showed the highest TNF-α levels, higher than the IC (*p* < 0.001), VID (*p* < 0.001) and VC (*p* = 0.020) groups.

Regarding CD163 immunostaining (Figure 4c) in the SR, the VOR, and VC groups showed higher levels compared to other groups. The VOR group had higher values than IC (*p* < 0.001) and VID (*p* = 0.004). In the same way this marker was higher in the VC group, compared to the IC group (*p* < 0.001) and VID (*p* < 0.001). A similar pattern was observed in the VA for this marker (Figure 4c). In this tissue, the VC group presented higher values than IC (*p* < 0.001) and VID (*p* < 0.001). Likewise, VOR group showed higher levels than IC group (*p* < 0.001) and VID (*p* < 0.001), although in this case VOR group levels were higher than in the SR. 

Calprotectin detection was higher in the IC group compared to the vaccinated groups in both SR (VID, VOR, and VC, *p* < 0.001 for all pairwise comparisons) and VA (VID, VOR, and VC, *p* < 0.001 for all pairwise comparisons) (Figure 4d). In addition, in the SR the VOR group showed higher levels of calprotectin than VID (*p* = 0.003) or VC (*p* < 0.001) groups. The same occurred in the VA, where the VOR group presented higher levels of calprotectin compared to VID (*p* = 0.022) and VC (*p* < 0.001). In the VA, the VID group presented higher levels of calprotectin compared to the VC group (*p* = 0.022).

#### 3.1.5. Interference with bTB

**Figure 5:**
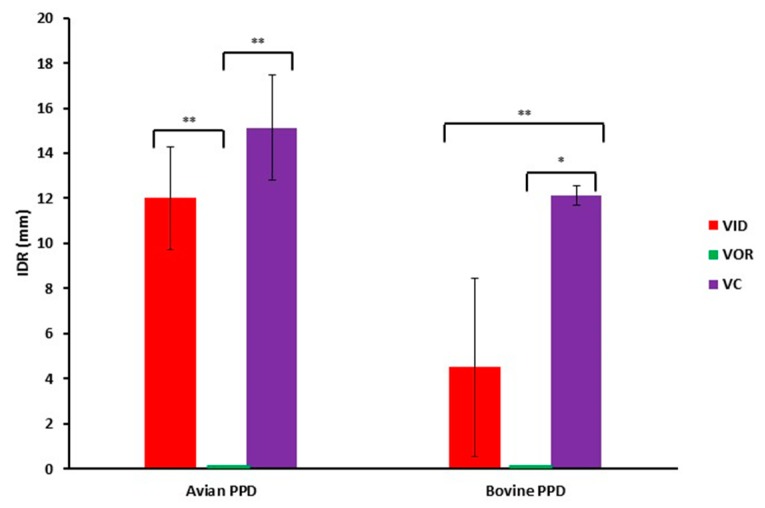
Mean skin reaction measured values to avian and bovine purified protein derivative (PPD) after sensitization in experimental intradermal vaccine (VID), experimental oral vaccine (VOR) and control vaccine (VC) animal groups. Error bars represent the standard error of the mean. * *p* < 0.01; ** *p* < 0.001.

The skin test interference analysis in guinea pigs revealed that VOR animals did not show skin reaction to A or B PPD, whereas VC and VID animals showed reactions for both tuberculins (Figure 5). VC animals presented significantly higher values compared to VID for the B PPD (*p* = 0.006). This test confirmed what was observed in rabbits in the sense that the orally administered experimental vaccine (VOR) did not trigger a detectable specific response either humoral or cellular, while the VC initiated both pathways. The VID, however, yielded a cellular immune response that could be related to the protection trend observed in the microbiological and histological parameters. 

## 4. Discussion

The present study aims to evaluate the effect of an experimental vaccination through different vaccination routes on MAP clearance as well as on the immune response. MAP exposed animals showed weight reduction compared to NIC animals, although significant differences were only observed with the VC group. This could be due to the added effect of MAP exposure by challenge and MAP antigens by vaccination.

At necropsy, gross pathology consisting of pale white spots corresponding to caseous necrosis in SR and VA, lymphangiectasia, and ileum and/or jejunum wall thickening were detected in 40%–80% of the challenged animals. Although significant differences were not observed among groups, the VID group presented the highest proportion of animals with no visible lesions, suggesting a higher protection capacity of intradermal delivery. Interestingly, the experimental vaccine applied through the intradermal route was also the vaccination strategy that presented the lowest proportion of MAP positive animal tissues evaluated by tissue PCR, indicating that the experimental vaccine administered through this route could be more effective at MAP clearance.

Regarding humoral response, only animals vaccinated with the commercial vaccine showed an increase in PPA-3 antibody titers during the experimental period. Humoral immune response against MAP has not been classically considered relevant, however some studies have suggested that this type of immune response could play a key role in mycobacterial infections [39,40,41]. In this context, antibody production induction by VC should not be excluded as one of the factors contributing to the protection exerted by this vaccine and demonstrated in previous studies in rabbits [34] cattle [13] and goats [24] where an increase in humoral response was reported concomitant with protection. Recently, it has been shown that the humoral response is essential for successful vaccine protection of PTB in sheeps with Gudair^®^, another inactivated whole cell vaccine [42]. As a fact, vaccination nodule size was positively correlated to antibody levels. VC did not contribute to complete bacterial clearance since MAP DNA was detected in 80% of the animals. However, this group did show a significant reduction in microscopic lesions compared to the IC group, which is important in terms of protection, as shown in previous studies [43]. In any case, anti-MAP antibody production can be a handicap for PTB diagnosis unless a differentiating infected from vaccinated animals (DIVA) serological assay is developed or a complementary test such as fecal screening is used. 

Macrophages are among the most abundant cell types in granulomas where they play a dual role, acting as bactericidal cells but also providing MAP with shelter [30]. Macrophages are increasingly recognized to represent heterogeneous cell populations with functional variability depending on polarization status. Following the Th1/Th2 classification system, the terms M1 and M2 were introduced into macrophage nomenclature to define the two main polarized activation states, where M1 macrophages would be pro-inflammatory and promoters of Th1 responses, while M2 macrophages would contribute to tissue repair and promote Th2 responses [44]. 

A recent study reports M1 macrophages as predominant in focal and multifocal forms in MAP infected cattle, suggesting the latent character of these type of lesions and the ability to control MAP infection, opposed to M2 macrophages that were the main type in diffuse multibacillary forms showing an immunoregulatory profile that would allow intracellular MAP growth [37]. Similarly, in human tuberculosis, M1 macrophages have been associated with early stages of infection where they promote the development of tuberculous granulomas and a protective response, while M2 macrophages would be associated with an advanced stage of the disease where host responses are less effective in controlling infection [45]. These studies address macrophage polarization associated to infection whereas this is the first report describing tissue macrophage polarization in MAP vaccinated and challenged animals. 

IFN-γ is one of the key cytokines in mycobacterial diseases and it induces M1 polarization of macrophages, leading to a pro-inflammatory response and increased microbicidal capacity. Macrophage bactericidal activity and phagocytosis are significantly enhanced by IFN-γ when it acts in concert with TNF-α, being necessary for *M. tuberculosis* control [46,47]. 

In the present study, higher IHC-scores for IFN-γ were observed in the SR of VID, VOR, and IC groups compared to VC animals, whereas higher scores for TNF-α were detected in the SR of VC and VID groups compared to VOR and IC groups. In addition, the VOR and VC groups showed a higher number of CD163 immunolabeled cells, meaning that lesions in those groups mainly present macrophages polarized towards M2. Taken all together, SR immunohistochemistry indicates that the VID group is the one showing a dominantly M1 polarized response, which is also compatible with the lower bacterial loads detected in the analyzed tissue.

In VA, IHC-scores for TNF-α were quite low in all infected groups. This could mean that there are functional differences between the intestinal areas, and that the VA lymphoid tissue exerts a less powerful or less protective immune response than SR, at least at this time point. CD163 scores were higher in the VOR and VC groups, meaning that these two vaccination schemes favor M2 polarization. These results are in agreement with the fact that all infected groups presented more severe lesions in the VA compared to the SR. 

It is worthy to mention that the IC group showed much higher IHC-scores for calprotectin in both SR and VA. Calprotectin is a major cytosolic protein complex present in monocytes that is expressed in tissue macrophages recently recruited from peripheral blood but whose expression is lost upon further differentiation [36]. This finding suggests that the IC group presented more active lesions while lesions in vaccinated groups seem to be under control and no new macrophages are incorporated into them. In this sense, some of the IC animals showed relatively high levels of IFN-γ and high levels of calprotectin compared to the vaccinated groups suggesting that vaccination is healing to some extent. A previous study has shown that MAP infected cattle tissues harbor a higher number of calprotectin immunolabeled cells in the diffuse forms compared to the focal ones and that IFN-γ was associated with focal and diffuse paucibacillary forms [48].

It must be highlighted that the immune response as well as macrophage polarization is a dynamic process and therefore interpretations based on analysis of data from one point may come short. Furthermore, experimental studies have shown that polarized macrophage phenotypes can be reversible both in vitro and in vivo [49,50]. Therefore, present results should be confirmed in a higher number of animals at different time points in order to fully characterize the effect of different vaccination routes on the local immune responses induced by the infection.

In addition to protection, a promising PTB vaccine candidate should also avoid interference with ante-mortem bovine tuberculosis diagnosis. Guinea pigs develop a strong delayed-type hypersensitivity response being a good model for tuberculin skin testing. In this sense, immunization in guinea pigs has shown promising results for oral vaccination with the experimental vaccine since it did not cause any skin test reactivity, although this should be confirmed and run with complementary assays proving immunization. The intradermal vaccination, however, caused some degree of skin test reaction that would be clearly differentiated with a comparative interpretation, but was completely negative in the ELISA by the end of the study. This means that the intradermal route could eventually combine protection against PTB with diagnostic differentiation (DIVA) both against bTB and PTB. Added to this, intradermal vaccination route minimizes lesions at vaccination site. These findings should be confirmed in a bovine trial and a proper candidate should show a balanced result in terms of high protection and low or null interference. 

## 5. Conclusions

In conclusion, PTB vaccination prior to challenge with MAP has a different effect on macrophage polarization compared to challenge with MAP alone. The administration route can influence the infection outcome and it can also have an impact on bTB diagnostic tests. In the present study, the experimental vaccine showed promising results when administered through the intradermal route, inducing higher bacterial clearances and a potential lower cross reactivity with the tuberculin skin test when compared to the commercial vaccine. Therefore, this vaccination route should be considered in future studies involving new immunological products against mycobacterial diseases. 

## Figures and Tables

**Table 1:** Immune markers detected in the immunohistochemistry analysis.

Antigen (Clone)	Specificity and Target Cells	Antigen Retreival Solution	Antibody Dilution	Reference
Bovine TNF-α (CC327)	Expressed in M1 macrophages.	6	1:200	[35]
Bovine IFN-γ (CC330)	Released by lymphocytes, macrophages and dendritic cells. Induces M1 macrophage polarization.	6	1:100	[36]
Human calprotectin (MAC387)	Expressed in activated, recently recruited macrophages among other cells.	9	1:200	[35]
Human CD163 (EDHu-1)	Expressed in M2 macrophages.	6	1:300	[35]

**Table 2:** Infection outcome in MAP infected rabbits.

		MAP PCR		Histopathology Score		
Group	ID	SR	VA	SR	VA	Gross Pathology Score
IC	37	+	−	2	2	2
	38	+	−	1	1	0
	40	−	+	3	3	4
	43	−	−	2	2	4
	44	+	+	2	4	0
VID	16	−	−	1	2	0
	17	−	−	2	1	0
	31	+	+	1	1	3
	32	−	+	2	2	0
	33	−	−	2	2	3
VOR	18	−	−	2	2	1
	19	−	+	2	2	1
	34	+	+	1	1	0
	35	+	−	1	2	3
	36	−	+	2	4	4
VC	14	−	−	1	1	0
	15	+	−	1	1	2
	28	−	+	1	1	1
	29	+	+	1	1	4
	30	+	+	1	2	3

## Supplementary Materials

The following are available online at https://www.mdpi.com/2306-7381/7/1/7/s1, Figure S1: Gross pathology observed in the digestive system. Figure S2: Vaccination inoculation sites, Figure S3: Macrophage polarization status.
