# Supplementary material for: Alternative Vaccination Routes against Paratuberculosis Modulate Local Immune Response and Interference with Tuberculosis Diagnosis in Laboratory Animal Models
Source: Vet Sci. 2020 Jan 10;7(1):7. doi: 10.3390/vetsci7010007 (PMC7157726; doi:10.3390/vetsci7010007)
Supplement: Supplementary file 1 [file vetsci-07-00007-s001.pdf]

veterinary sciences

MDPI

Article

Alternative Vaccination Routes against Paratuberculosis Modulate Local Immune Response and Interference with Tuberculosis Diagnosis in Laboratory Animal Models

Rakel Arrazuria [1], Iraia Ladero [1], Elena Molina [1], Miguel Fuertes [1], Ramón Juste [1], Miguel Fernández [2], Valentín Pérez [2], Joseba Garrido [1] and Natalia Elguezabal [1,*]

[1] Animal Health Department, NEIKER-Instituto Vasco de Investigación y Desarrollo Agrario. Derio, E-48160 Bizkaia, Spain; raarrazu@utmb.edu (R.A.); iladero@neiker.eus (I.L.); emolina@neiker.eus (E.M.); mfuertes@neiker.eus (M.F.); rajuste@serida.org (R.J.); jgarrido@neiker.eus (J.G.)

[2] Department of Animal Health, Instituto de Ganadería de Montaña (CSIC-ULE), Facultad de Veterinaria, Universidad de León, E-24071 Leon, Spain; m.fernandez@unileon.es (M.F.); valentin.perez@unileon.es (V.P.)

* Correspondence: nelguezabal@neiker.eus; Tel.: -34-94-403-4300

Received: 15 November 2019; Accepted: 8 January 2020; Published: 10 January 2020

Supplementary Materials

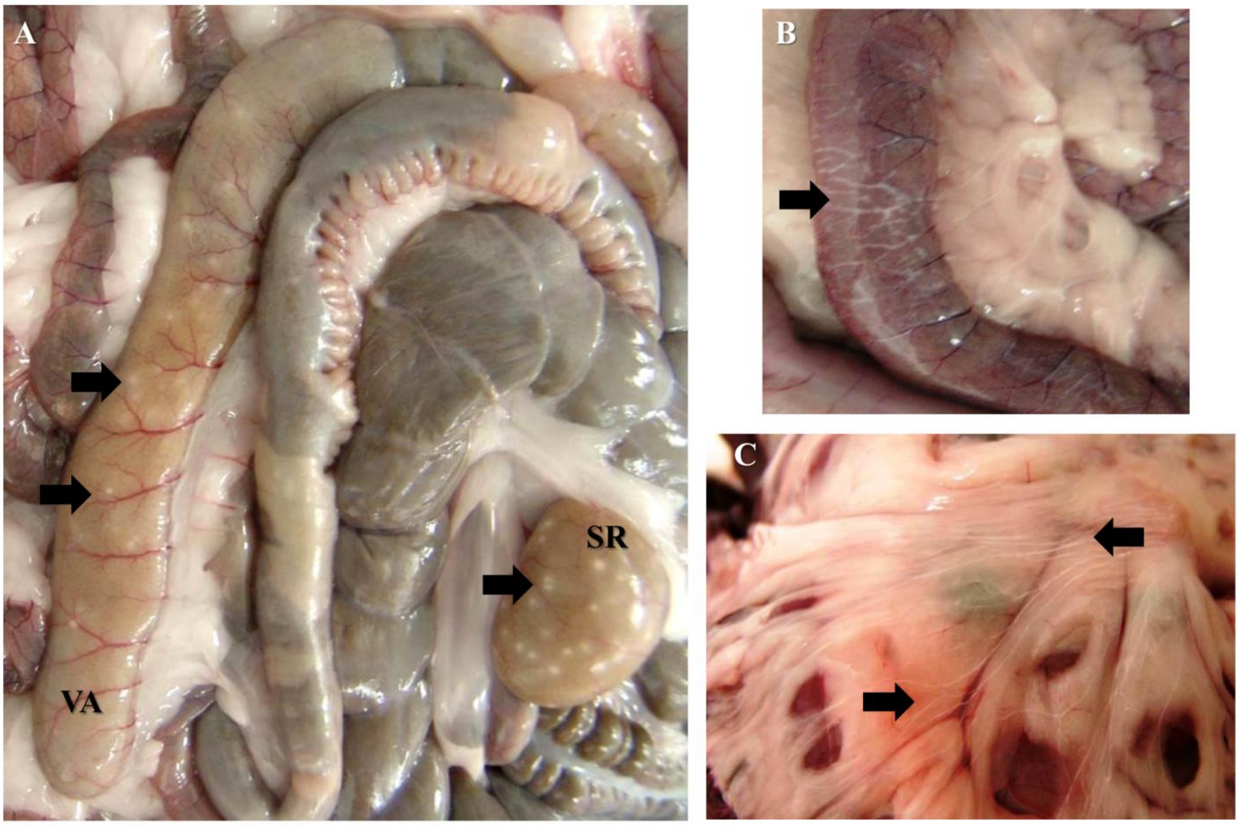

Figure S1. Gross pathology observed in the digestive system. (**A**) Pale white spots in sacculus rotundus (SR) and vermiform appendix (VA). (**B**) Dilatation of lymphatic vessels (lymphangiectasia) of intestinal serosa. (**C**) Lymphangiectasia in the mesentery.

veterinary sciences

MDPI

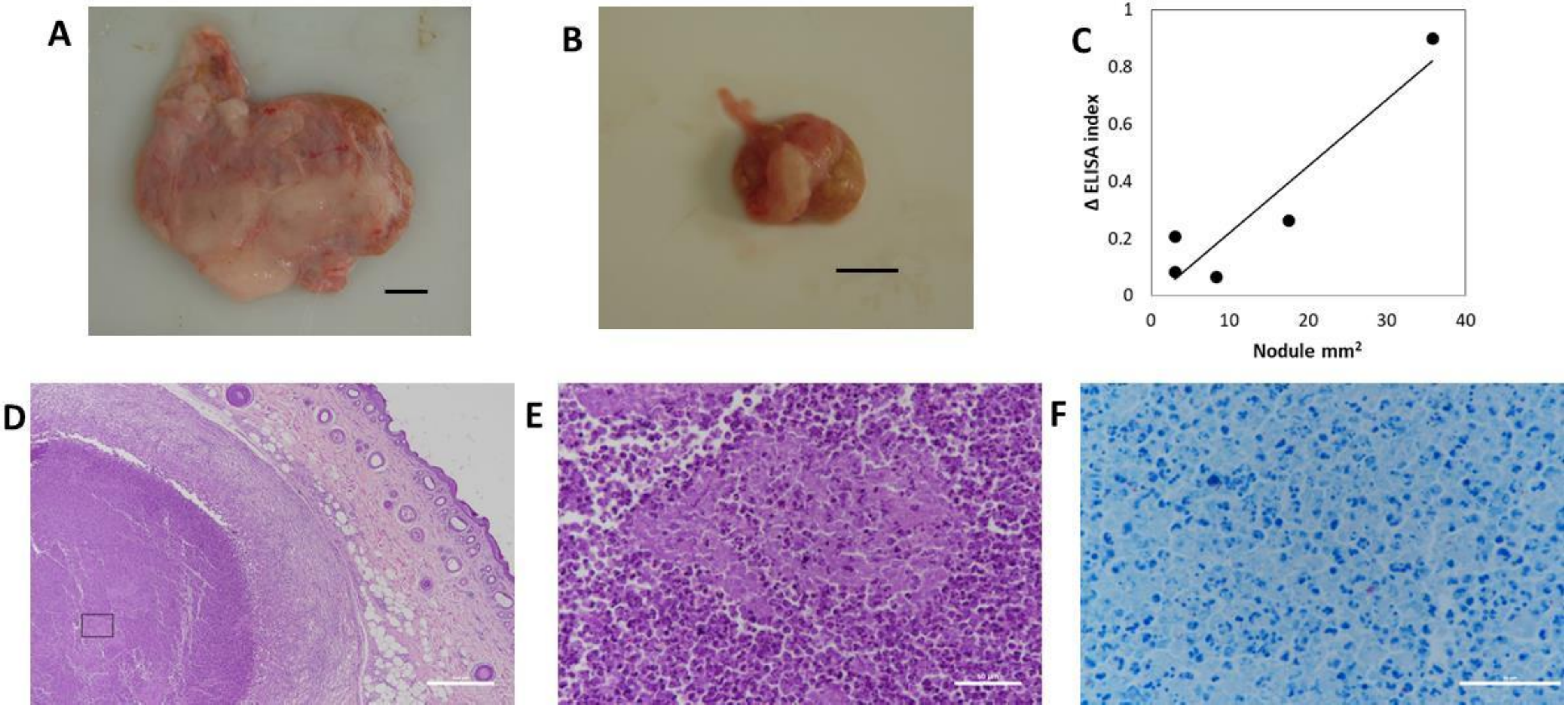

Figure S2. Vaccination inoculation sites. (**A**,**B**) representative photographs of nodule formed in subcutaneously vaccinated rabbits (bars are equivalent to 1 cm), (**C**) dot plot showing correlation between vaccination nodule and ELISA index (S6–S0) in the subcutaneously vaccinated group (Pearson: 0.935, *p*: 0.02). The trend line represents the least square estimation. (**D**) Cutaneous pyogranuloma in intradermic vaccination site on guinea pig (haematoxilin-eosin staining; bar = 500 µm). (**E**) Magnification of (**D**), necrosis and neutrophil inflammatory infiltrate in intradermic injection site (haematoxilin-eosin staining; bar = 50 µm). (**F**) Acid-fast bacilli in necrotic areas of intradermic injection site (Ziehl-Nelsen staining; bar = 50 µm).

veterinary sciences
MDPI

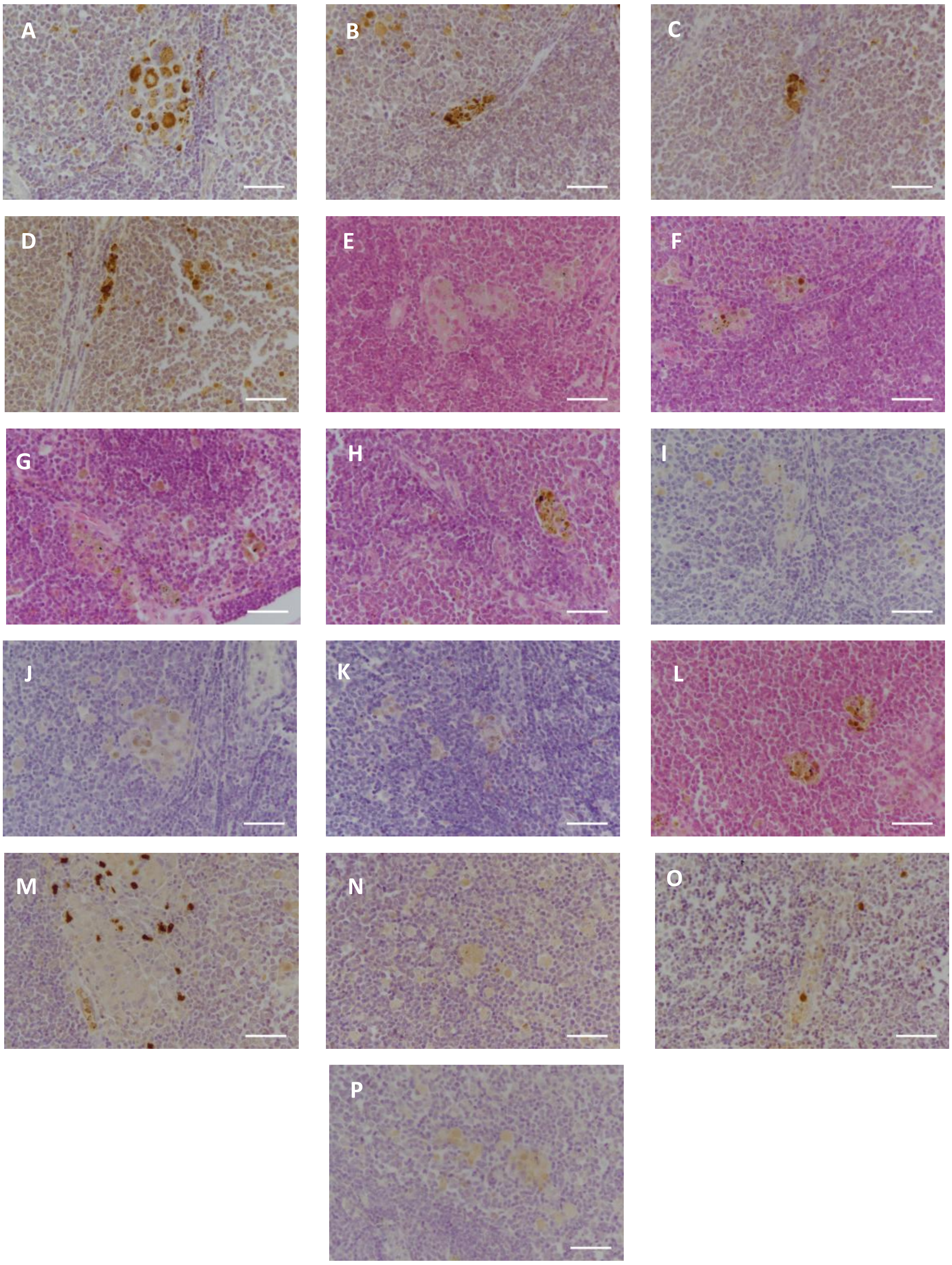

Figure S3. Macrophage polarization status. Representative figures of the immunostaining in sacculus rotundus for each marker and experimental group (bar = 50 µm). (**A**–**D**) IFN-γ, (**E**–**H**) TNF-α, (**I**–**L**) CD163 and (**M**–**P**) calprotectin. A, E, I and M are infected controls (IC); B, F, J and N are experimental intradermal vaccine animals (VID); C, G, K and O are experimental oral vaccine animals (VOR), D, H, L and P are control subcutaneous vaccine animals (VSC). Bars in micrographs represent 50 µM.
